# Real world preventative drug management of migraine among Spanish neurologists

**DOI:** 10.1186/s10194-019-0971-6

**Published:** 2019-02-15

**Authors:** D. García-Azorin, S. Santos-Lasaosa, A. B. Gago-Veiga, J. Viguera Romero, A. L. Guerrero-Peral

**Affiliations:** 1 Headache Unit, Neurology Department, Hospital Universitario Clínico de Valladolid, Avda. Ramón y Cajal 3, 47005 Valladolid, Spain; 2 Headache Unit. Neurology Departmente, Hospital Clínico Universitario Lozano Blesa, Zaragoza, Spain; 3 Headache Unit, Neurology Department, Hospital Universitario de la Princesa, Madrid, Spain; 4 Headache Unit, Neuroscience Department, Hospital Universitario Virgen Macarena, Sevilla, Spain; 5 Institute for Biomedical Research of Salamanca, IBSAL, Salamanca, Spain

**Keywords:** Primary headache, Migraine, Preventive treatment, onabotulinumtoxinA, Topiramate

## Abstract

**Background:**

Many different preventatives have showed efficacy in the treatment of migraine. National guidelines differ in their recommendations and patients’ characteristics are usually taken into account in their selection. In Spain, real life use of preventive therapies seems to be heterogeneous. We aimed to evaluate differences in clinical practice and adherence to national guidelines among Spanish neurologists.

**Methods:**

Observational descriptive study. A survey was conducted among neurologists ascribed to the Spanish Society of Neurology. Participants were differentiated in accordance with their dedication to headache disorders. We analysed socio-demographic parameters and evaluated 43 questions considering migraine management as well as therapeutic choices regarding migraine sub-types and finally, neurologists’ personal perception.

**Results:**

One hundred fifty-five neurologists participated from 17 different regions, 43.4% of them female and 53.3% under 40 years of age. 34.9% confirmed headache disorders as their main interest.

The first choice for preventive therapy in chronic migraine among participants was topiramate (57%) followed by amytriptiline (17.9%) and beta-blockers (14.6%). However in episodic migraine, the preferred options were beta-blockers (47.7%), topiramate (21.5%) and amytriptiline (13.4%). Regarding perceived efficacy, topiramate was considered the best option in chronic migraine (42.7%) followed by onabotulinumtoxinA (25.5%) and amitryptiline (22.4%). Where episodic migraine was concerned, surveyed neurologists perceived topiramate (43.7%) and beta-blockers (30.3%) as the best options. When we evaluated the duration of treatment use with a view to adequate therapeutic response, 43.5% of neurologists preferred 3 months duration and 39.5% were in favour of 6 months duration in episodic migraine. However, considering the preferred duration of treatment use in chronic migraine, 20.4% recommended 3 months, 42.1% preferred 6 months and 12.5% and 22.4% opted for 9 and 12 months respectively. When considering onabotulinumtoxinA therapy, the number of prior therapeutic failures was zero in 7.2% of neurologists, one in 5.9%, two in 44.1%, three in 30.9% and four or more in 11.9%. Following an initial treatment failure with onabotulinumtoxinA, 49% of subjects decided against a second treatment. The number of OnabotA procedures before considering it as ineffective was two in 18.9% of neurologists, three in 70.8% and four in 10.4%.

**Conclusions:**

The initial management of migraine among Spanish Neurologists is in line with most guidelines, where first choice preventative drugs are concerned. The Management of episodic migraine differed from chronic migraine, both in terms of neurologist preference and in their perceived efficacy.

**Electronic supplementary material:**

The online version of this article (10.1186/s10194-019-0971-6) contains supplementary material, which is available to authorized users.

## Introduction

Headache is the most frequent reason for referral to outpatient neurology offices in Spain [1]. Headache is a common presenting complaint in neurology, migraineurs being the most common subgroup in the outpatient setting [2]. The Spanish Society of Neurology periodically publishes guidelines on the main neurological conditions, in order to help clinicians in their daily practice.

The Spanish Headache Study Group guidelines [3] recommended topiramate and beta-blockers as the first choice of preventive therapy in Episodic Migraine, followed by flunarizine, Lisinopril and candesartan. Valproic acid is recommended only in a tertiary referral centre and is currently contraindicated in women of childbearing age [4]. On the other hand, the first oral preventive option in Chronic Migraine is topiramate, followed by amitriptyline and in a third level flunarizine and beta-blockers.

Despite the high number of therapies available, some of them have not been properly studied and the evidence is based on experts’ opinions [5]. The arrival of new drugs to our armamentarium should improve the care of migraine patients [6, 7]. Understanding the needs of clinicians may help in the development of new therapeutic strategies and guidelines.

We aimed to evaluate the preferences in the management of migraine with preventive therapies, the perceived efficacy and safety of the current therapies among Spanish Neurologists.

## Material and methods

We conducted an observational, transversal study. The studied population was the community of Spanish Neurologists ascribed to the Spanish Society of Neurology.

The study included an anonymous survey mailed three times to all neurologist members of the Society. It included 8 questions about demography and 43 concerning preventative preferences and utilization.

The survey analysis considered if the neurologist was a member of the Spanish Headache Study Group (SHSG) and those in whom headache disorders were their main area of interest. For analytic purposes, we categorized responders in two differentiated age groups: junior neurologists if they were under 40 years old (juniors) and senior neurologists if they were 40 years old or above (seniors in advance). In Spain most trainees complete their Neurology training at age 29 following 4 years of residency.

Demographic variables were gender, age group, region of origin, main area of interest, adscription to the SHSG, and duration spent working in headache medicine.

Eligibilty criteria of treatments included all those mentioned in the Official Guidelines of the Spanish Headache Study Group, namely: beta-blockers, amitriptiline, topiramate, valproic acid, zonisamide, lisinopril, candesartan, fluoxetine, venlafaxine, desvenlafaxine, lamotrigine, magnesium, flunarizine, riboflavine, pregabalin, onabotulinumtoxinA (onabotA) and anaesthetic blockade of greater occipital nerves (GON) [3]. We analysed drugs individually, including two specific questions about the percentage of combined treatments in the first visit and in global.

We specifically evaluated which was the preferred beta-blocker and we included among the possible answers propranolol, nadolol, atenolol, bisoprolol, nebivolol and esmolol. We analyzed by separate onabotA management in terms of number of prior preventive failures, employed units per procedure and long-term management.

Surveyed neurologists were asked about their preferred first and second choices in preventive therapy in chronic migraine (CM), episodic migraine with aura (EMWA), and in episodic migraine without aura (EMOA). We also gave special consideration to conditions such as depression or pregnancy.

Participants were also questioned about their personal opinion regarding the most effective drugs in both Chronic and Episodic Migraine and which they thought were the best-tolerated drugs.

The Scientific Committee of the Spanish Society of Neurology approved the study and all the participants agreed to participate voluntarily.

We present data as number and percentage. Missing data was managed by complete case analysis. We employed SPSS v16.0 for the Statistical Analysis. For the comparison of qualitative variables, we used Chi [2] test. In the comparison of continuous variables with qualitative variables, the employed test was Student t test and Median test in case of non-normal distribution or < 30 variables per group. Correlation between quantitative variables was analysed with Pearson test. We considered an alpha error value of 0.05.

## Results

### Demographic parameters

We received responses from 153 neurologists, among which 53 (34.9%) were ascribed to the Spanish Headache Study Group. The percentage of female participants was 43.4%. Age of participants was < 30 years in 11.8%, 30–39 in 41.4%, 40–49 in 16.4% and > 50 in 30.4%.

The most frequent regions were Madrid, with 32 participants (21.1%) and Catalonia with 24 (15.8%). The time since they were focused on headache disorders was < 6 years in 35.4%, 6–10 years in 14.6%, 11–15 years in 14.6%, 16–20 in 10.4% and > 20 years in 25%.

### First choice drugs

#### First choice in chronic migraine

Topiramate was the first choice drug in 57% of responders, followed by amitriptyline (17.8%), beta-blockers (14.6%) and flunarizine (6%). The most frequent second choice drug were beta-blockers (25.7%) followed by topiramate (23.2%), amitriptyline (20.5%), onabotA (10.6%) and flunarizine (9.3%). Complete data on all possible drugs is shown in the Additional file 1.

Management of CM seemed to be different among general neurologists when compared with those focused on headache disorders, particularly concerning the first choice selection of topiramate (49.5% vs. 71.2%, p:0.017), amitriptyline (23.2 vs. 7.7%, p:0.016) and flunarizine (9.1% vs. 0%, p:0.027).

Comparison of juniors and seniors did not yield any significant differences.

#### First choice in episodic migraine without aura

The preferred drugs were beta-blockers (47.7%), followed by topiramate (21.5%), amitriptyline (13.4%) and flunarizine (11.4%). The most frequent second choice was topiramate (44.6%), followed by beta-blockers (23.6%), amitriptyline (15.5%), flunarizine (9.5%) and zonisamide (4.1%). Further data is available in Additional file 1.

#### First choice in episodic migraine with aura

Topiramate was the first choice for 50.3% of participants, followed by beta-blockers (23.2%) amitriptyline (9.3%), flunarizine (6.6%) and lamotrigine (2.6%). The most frequent second choice was topiramate (31.8%), beta-blockers (25.7%), lamotrigine (10.1%), amitriptyline (9.5%), zonisamide (8.8%), flunarizine (6.8%) and valproic acid (6.1%). Full list of responses are available in the Additional file 1.

We did not find significant differences between headache specialists and general neurologists concerning the preferred drug in episodic migraine treatment, neither with nor without aura.

Between junior and seniors, there was a trend but results did not reach statistical significance, to use less topiramate among juniors (17.7% vs. 25.7%, p:0.1) and flunarizine (8.9% vs. 14.3%, p:0.2) and higher use of amitriptyline (16.5% vs. 10.0%, p:0.2).

**Fig. 1:**
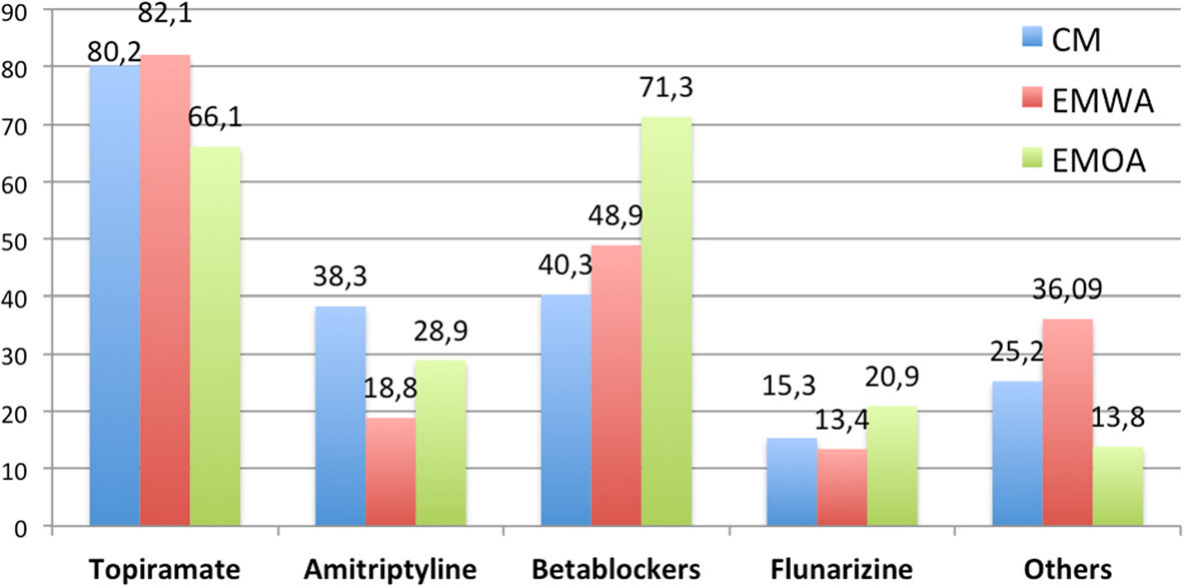
First choice drugs in Chronic Migraine (CM) (blue), Episodic Migraine With Aura (EMWA) (red) and Episodic Migraine Without Aura (EMOA) (green). Percentage shows the pooled percentage of people who responded each drug as first or second choice in each indication

Figure 1 represents the pooled first and second choices in Chronic Migraine (CM), Episodic Migraine with aura (EMWA) and Episodic Migraine without aura (EMOA).

Management was described to be different when treating female patients in 76.3% of neurologists. The preferred option in case of pregnancy was beta-blockers in 57.7%, magnesium (17.3%), Greater Occipital Nerve blockades (16.3%), onabotA (3.8%), lamotrigine (2.9%) and others (2%). Valproic acid was avoided in female migraineurs by 90.1% of responders.

In patients with depression, the preferred drugs were amitriptyline in 67.1%, venlafaxin in 15.3%, and topiramate in 11.3%.

### Responses about perception of efficacy

#### Chronic migraine

Topiramate was considered the most effective drug in the treatment of CM by 42.7% of responders, followed by onabotA in 25.2%, amiptriptyline (22.4%), beta-blockers (3.5%) and flunarizine (2.1%).

The second most frequently considered drug was also topiramate in 34.3%, followed by amitriptyline (19.7%), beta-blockers (15.3%), onabotA (8%), venlafaxin (5.8%), valproic acid and zonisamide (5.1% each). Full list of responses can be consulted in Additional file 1.

OnabotA was considered as the most effective drug more frequently among neurologists with special interest in headache disorders than in general neurologists (34% vs. 18.2%, p:0.029). In the group of general neurologists, amitryptiline tended to be considered as the most effective drug in more cases than in headache specialists (27.3% vs. 9.4%, p:0.1). We did not found statistically significant differences between junior and senior neurologists concerning preferences in terms of efficacy.

#### Episodic migraine

The drug perceived as most effective in episodic migraine was topiramate (43.7%), followed by beta-blockers (30.3%), amitriptyline (14.8%) and flunarizine (8.5%). Beta-blockers were considered the second most effective option in 32.9% of the answers, followed by topiramate (28.6%), amitriptyline (17.1%) and flunarizine (11.4%). Full data about efficacy can be consulted in Additional file 1.

Preferences of neurologists with special interest of headache did not differed from general neurologists, only amitriptyline was described as less effective (17.6% vs. 25.0%, but differences were not statistically significant p:0,56).

We found differences in the perception of efficacy of topiramate and amitriptyline comparing senior and junior neurologists. Seniors considered topiramate as the most effective more often (54.5%vs34.2%, p:0,022), whereas juniors considered amitriptyline as the most effective in more cases (21.1%vs7.6%, p:0,033).

The preferred beta-blocker among surveyed neurologists was propranolol (62.1%), followed by nadolol and nebivolol (15.85% each), atenolol (4.1%) and bisoprolol (2.1%).

### Perceptions about tolerability

Drugs considered as best tolerated in a young patient without any comorbidity were betablockers (42.4%), followed by flunarizine (14.6%), onabotA (11.3%), topiramate and amitriptyline (9.3% each), and magnesium (4%). Full data about tolerability preferences can be seen in Additional file 1.

**Fig. 2:**
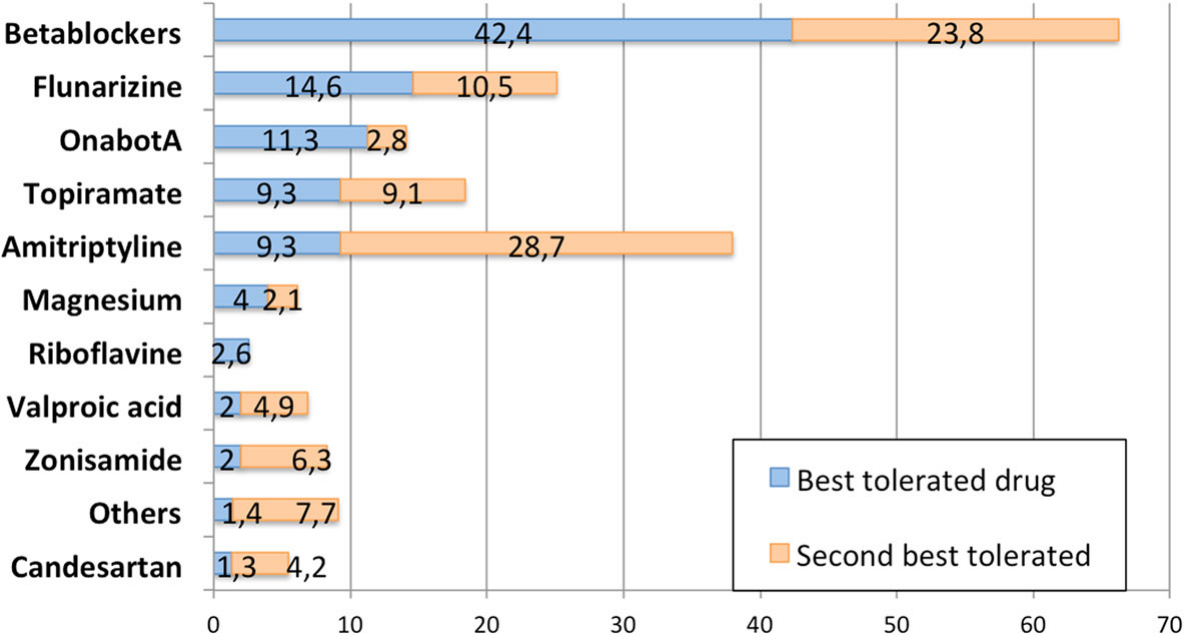
Best tolerated drugs according to responders’ opinions. In light blue, percentage of responders that selected each drug as the best tolerated. In light orange, percentage of responders that selected each drug as the second best tolerated. Number represents percentage

Figure 2 shows survey responses considering tolerability, showing the percentage of neurologists that considered each drug as the best tolerated and the second best tolerated respectively.

**Fig. 3:**
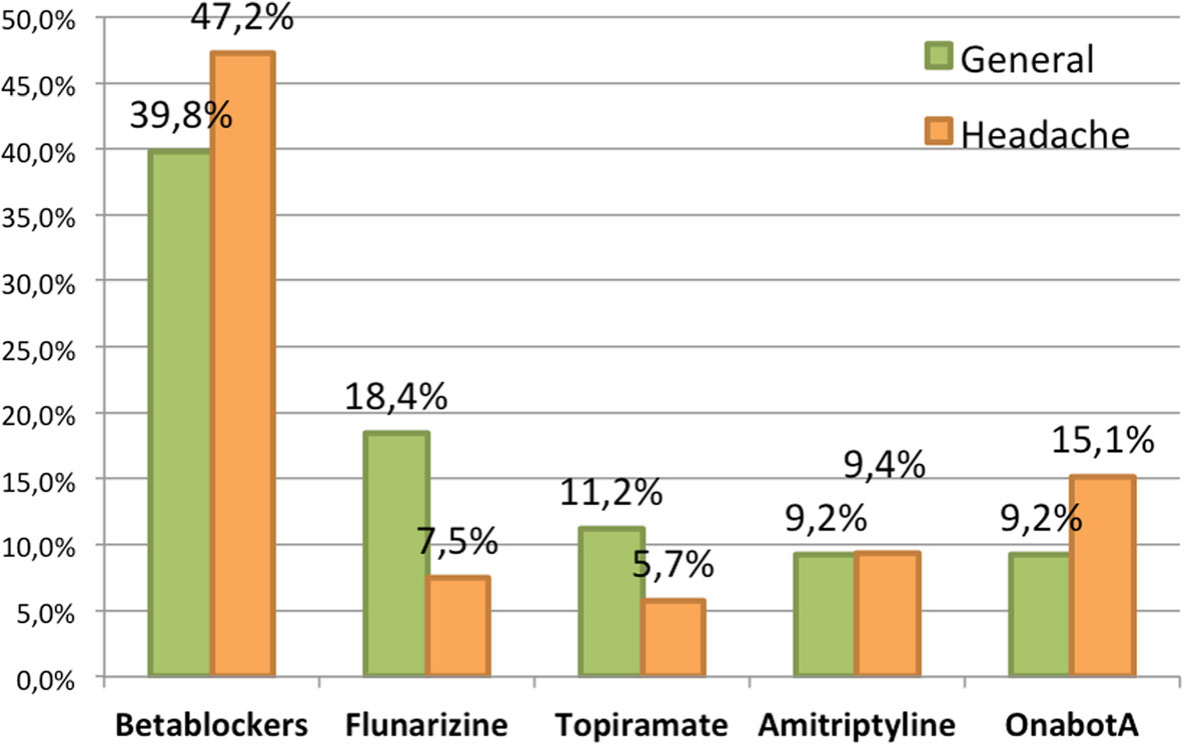
Opinions among surveyed neurologists about each drug tolerability. In orange: answers from Neurologists with special interest in headache. In green: answers from general neurologists

Differing opinions emerged between headache specialists and general neurologists where the best-tolerated drugs were concerned, however these results were not statistically significant Fig. 3.

In the correlation analysis we found that the correlation between considering topiramate well tolerated and selecting it as first choice in CM was r = 0,95, (*p* = 0,24, Pearson test), in EMOA was r = 0,23 (*p* = 0,005) and in EMWA r = 0,23 (*p* = 0,005); whereas the correlation between considering it as the most effective and selecting it as first choice was higher, r = 0,46 in EMOA (r < 0,0001) and r = 0,43 in EMWA (*p* < 0,0001) and for CM was r = 0,31 (*p* < 0,0001).

### Management of patients

Participants described patients comorbidities as the most important factor when choosing a preventative (70.2%) followed by guidelines recommendations (13.9%), personal experience (11.3%) and patients’ preferences (4.6%).

Regarding the management in primary care, 80.9% of the surveyed neurologists considered that the general practitioner should prescribe preventatives before referral to neurology office. The number of preventatives considered to fail before referring the patient was none in 4.6%, one in 13.8%, two in 56.6% and three or more in 25% for episodic migraine whereas in chronic migraine was none in 21.1%, one in 22.4%, two in 40.8% and three or more in 15.8%.

**Fig. 4:**
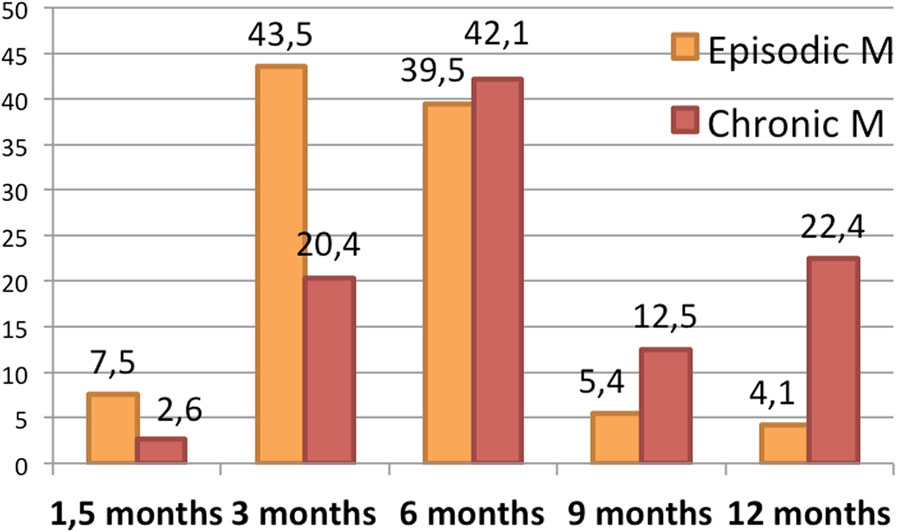
Optimal duration of preventative treatment according to surveyed neurologists. Numbers represent the percentage of responders that answered each duration. Orange bars represent episodic migraine; red bars represent chronic migraine

Regarding the duration of preventive therapy until achieved therapeutic response, in EM 51% of responders affirmed to keep the treatment for 3 months or less whereas in CM 42,1% treated during 6 months and 34,9% during at least 9 months. Figure 4 shows the duration of the treatment in EM or CM.

Polytherapy was only considered in selected cases in the first visit by 22.3% of the participants. When asked about management in their patients, the percentage of them treated with polytheraphy was estimated to be 0–10% by 14.5% of neurologists, 11–25% in 44.1%; 26–50% in 27.6%; 51–75% in 9.2% and > 76% in 4.6%.

### OnabotulinumtoxinA management

Regarding specific OnabotA questions, we asked about the number of failure (efficacy or tolerability) in oral preventatives before starting OnabotA. Response was none in 7.2% of neurologists, one in 5.9%, two in 44.1%, three in 30.9%, four in 9.9% and five or more in 2%. When asked whether they considered specifically the failure to any preventative, 51.4% mentioned topiramate, 4.9% amitriptyline, 4.2% beta-blockers and 36,6% said they did not consider any specific failure.

**Fig. 5:**
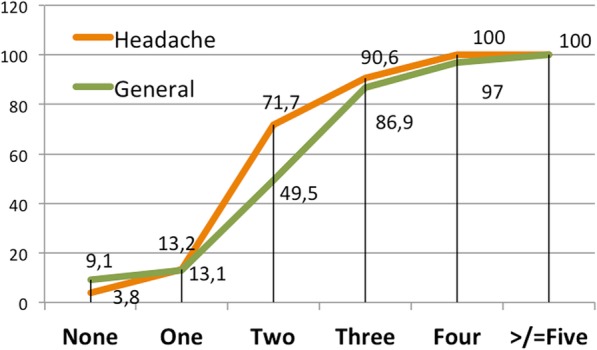
Number of oral preventative failures prior to the OnabotA therapy. Numbers represent the percentage of responders that affirmed to start OnabotA after the failure of each number of therapeutic failures. In orange, answers from Headache Specialists; Green, answers from general neurologists

When comparing between headache specialists and general neurologists, the former started OnabotA earlier than general neurologists (p:0.014) Fig. 5.

We did not find differences when comparing juniors and seniors and 54.3% affirmed to start OnabotA after two preventive failures and 83,9% after the failure of three preventive drugs (p:0.49).

**Fig. 6:**
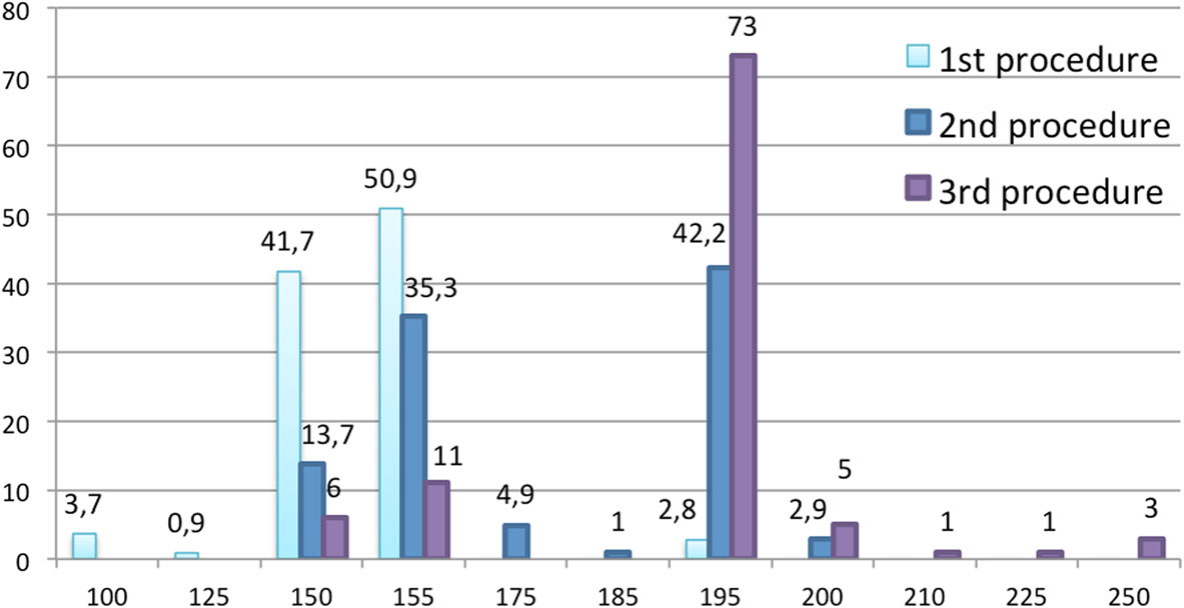
Percentage of participants that administer each defined number of OnabotA units per procedure. Light blue represents the first procedure, dark blue the second procedure and purple the third procedure

Concerning OnabotA dose, in the first procedure, 50.9% of responders injected 155 Units and 41.7% 150 U. Figure 6 shows the units used per procedure by the surveyed neurologists. Following an initial treatment failure, 51% of responders increased the dose in the second procedure and in case of inefficacy, 83% of responders did increased the dose from 155 units in the third procedure.

The number of OnabotA procedures before considering it as ineffective was two in 18.9% of neurologists, three in 70.8% and four in 10.4%.

Only 66 participants (38,2%) described to be assisted by a nurse in the OnabotA preparation. The percentage of clinicians that self-charged the medication was higher among headache specialists compared with general neurologists (50.9 vs. 39.4%, p:0.008).

**Fig. 7:**
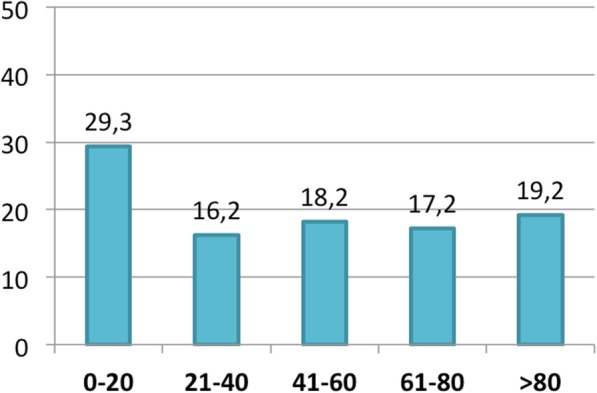
Discontinuation trial of OnabotA in case of efficacy: percentage of cases in which responders **try** to discontinue OnaboA in case of efficacy

**Fig. 8:**
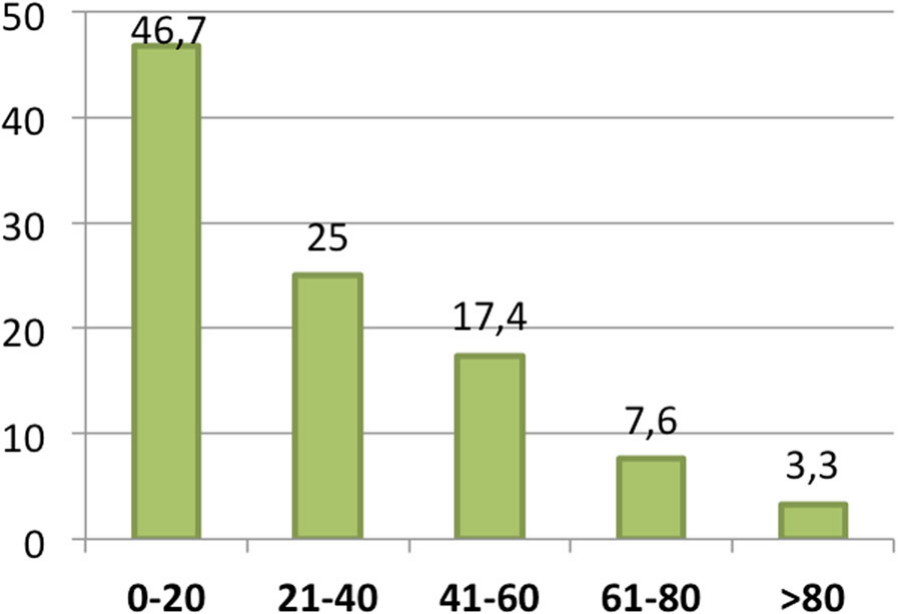
Successful discontinuation of OnabotA in case of efficacy: percentage of cases in which responders **succeed** to discontinue OnaboA in case of efficacy

Figure 7 shows the percentage of patients in which responders affirmed to perform a reduction of OnabotA in case of efficacy, trying to stop the therapy and Fig. 8 shows the percentage in which they were able to finally stop it.

## Discussion

### First choice

The main findings of our study were that topiramate was selected as the first choice drug in CM and EMWA and the second choice in EMOA, whilst beta-blockers were the drugs of choice in EMOA. These findings follow the Spanish Headache Study Group recommendations [3], but differences were found in the second and third choice, as few responders considered valproic acid as a potential option and amitriptyline was selected before flunarizine in all three groups.

According to European Medicines Agency [4] recommendations, subscribed by the European Headache Federation, valproic acid should be avoided in women in childbearing age. In our sample, 90.1% of responders followed that recommendation.

The percentage of responders that mentioned other drugs such as candesartan [8], lisinopril [9], zonisamide [10], lamotrigine [11] was around 10%, despite our guidelines place some of them on a par with amitriptyline.

### Efficacy

Only topiramate and OnabotA have proved their efficacy in randomized controlled trials in Chronic Migraine and only recently its efficacy has been directly compared [12]. Despite the different methodology of the studies, number of headache days reduction in the pivotal trials ranged from − 3,5 to − 6,4 for topiramate [13, 14]and − 7,8 to − 9 for OnabotA [15, 16]. Our findings suggest that neurologists perceived Topiramate as more effective than OnabotA, both among headache specialists and general neurologists. In the last group, perception about OnabotA efficacy was even lower and amitriptyline was chosen as more efficacious.

We also consider of interest how beta-blockers were perceived, as they were considered as the second most effective option in EM, slightly after topiramate. However, in Chronic Migraine, the percentage of neurologists that considered them as the most effective option was significantly lower, only 3.5% them in comparison with 30.3% in EM. Only one study compared them directly and could not find statistically significant differences in Episodic Migraine patients [17].

Another topic of discussion is the duration of the preventive treatment. Spanish guidelines recommend at least 6 months of treatment [3], but we found a significant heterogeneity. We should harmonize the minimal duration, how much should we increase the dose in case of lack of tolerability and how long should we keep the treatment before considering it a treatment failure.

### Tolerability

Concerning the tolerability profile, most of the responders preferred beta-blockers but the preferred drugs were flunarizine, OnabotA, amitriptyline and topiramate. Some of these drugs have been associated with adverse events in up to 75 to 82,5% of the patients in the pivotal trials [13, 14].

Despite the difference of administration, OnabotA was perceived as well tolerated by a high percentage of neurologists, in line with long-term studies show [18], and it was selected before GON blockades.

### OnabotulinumtoxinA

Spanish experts recommendations for the use of OnabotA stated that the first procedure should be performed according to the PREEMPT paradigm, administering 155 Units and in case of lack of response, dose could be increased up to 195 Units in the first three procedures [19]. We found that in certain cases, neurologists admitted to using lesser or higher doses.

To date, some factors have been associated with an increased efficacy to OnabotA in CM: use of higher OnabotA dose up to 195 units [15–19], a shorter evolution of Chronic Migraine, a lesser number of oral preventatives prior to OnabotA using [20]. Despite that Spanish Guidelines recommend to consider OnabotA after two therapeutic failures and Spanish Health Care System covers OnabotA costs all across the country, only 49.5% of general neurologists surveyed admitted to starting it after the failure of two preventatives. Similar data in an Italian survey, showed that only 39,7% of responders said they commenced OnabotA before the failure of 3 preventatives [21]. In our sample, it was surprising that one out of five responders did not increase the dose up to 195 units at the third procedure in case of lack of response. Publication of European guidelines [22, 23] should encourage clinicians to consider it when indicated as it has proven to be an effective therapy also in real world studies and increase properly the dose in case of lack of response [20].

### Future perspectives

The fact that all currently available preventive drugs have been developed for other indications may be related to the presence of adverse events and poor compliance. The arrival of novel specific drugs such as anti-CGRP antibodies and gepants could dramatically change the clinical picture [24, 25]. Nevertheless, despite most of the participants said that the presence of comorbidities was the main factor in the selection of the therapy; we found that the decision was correlated most with the perceived efficacy rather than the tolerability profile.

The panorama will change in the coming years and some factors will be of striking importance, such as the availability of the therapies, its efficacy in real world setting and the diffusion among the majority of clinicians. We support the creation of new European Guidelines and the harmonization of the National Guidelines according to the current literature.

Our study has some limitations. As an online survey, not all the members of the Headache Study Group responded to it and the answers might not represent the opinions of all the Headache Specialists. Among the 51 questions, some of them were subjective and we did not allowed more than two possible answers.

Also, our results reflect only opinion among Spanish neurologists, with a public healthcare system different of those from many other European Countries. Finally, we did not analysed non-pharmacological therapies or comorbidities management, which should be considered for future studies.

## Conclusion

Our study states that the main criterion in the selection of treatments is the subjective perceived efficacy, topiramate being the drug considered as the most effective and therefore the first choice drug in CM and EMWA.

Despite the availability of many novel therapies, most of the clinicians employed the classical drugs.

## Additional file

Additional file 1: **Table S1.** First and second choices in chronic Migraine. Number represent percentage or the total responses. **Table S2.** First and second choices in episodic migraine without Aura. Numberrepresentspercentage of total responses. **Table S3.** First and second choices in episodic migraine with Aura. Numbers represents percentage of total responses. **Table S4.** Perception about efficacy of the different drugs in the treatment of chronic migraine. Numbers represent percentage of the answers. **Table S5.** Perception about efficacy of the different drugs in the treatment of episodic migraine. Numbers represent percentage of the answers. **Table S6.** Answers about the drugs considered as the best tolerated and second best tolerated. (DOCX 68 kb)

## Abbreviations

CM: Chronic migraine
EMOA: Episodic migraine without aura
EMWA: Episodic migraine with aura
Juniors: Neurologists younger than 40 year old
Onabot A: OnabotulinumtoxinA
Seniors: Neurologists aged 40 or more
SHSG: Spanish Headache Study Group
