# Supplementary material for: Real world preventative drug management of migraine among Spanish neurologists
Source: J Headache Pain. 2019 Feb 15;20(1):19. doi: 10.1186/s10194-019-0971-6 (PMC6734505; doi:10.1186/s10194-019-0971-6)
Supplement: Supplementary file 1 — Table S1. First and second choices in chronic Migraine. Number represent percentage or the total responses. Table S2. First and second choices in episodic migraine without Aura. Numberrepresentspercentage of total responses. Table S3. First and second choices in episodic migraine with Aura. Numbers represents percentage of total responses. Table S4. Perception about efficacy of the different drugs in the treatment of chronic migraine. Numbers represent percentage of the answers. Table S5. Perception about efficacy of the different drugs in the treatment of episodic migraine. Numbers represent percentage of the answers. Table S6. Answers about the drugs considered as the best tolerated and second best tolerated. (DOCX 68 kb) [file 10194_2019_971_MOESM1_ESM.docx]

**Real world preventative drug management of Chronic Migraine among Spanish Neurologists**

**ABSTRACT:**

**Background:**

Many different preventatives have showed efficacy in the treatment of migraine. National guidelines differ in their recommendations and patients’ characteristics are usually taken into account in the selection. In Spain, real life using of preventive therapies seem to be heterogeneous. We aimed to evaluate differences in clinical practice and adherence to national guidelines among Spanish neurologists.

**Methods:**

Observational descriptive study. A survey was conducted among neurologists ascribed to the Spanish Society of Neurology. Participants were differentiated considering if they were mainly dedicated to headache disorders. We analyzed socio-demographic parameters and we evaluated 43 questions considering migraine management, and therapeutic choices regarding migraine sub-types and neurologists´ personal perception.

**Results:**

152 neurologists participated from 17 different regions, 43.4% of them female and 53.3% younger than 40 years. 34.9% affirmed to be mainly interested in the headache field.

The first choice for preventive therapy in chronic migraine among participants was topiramate (57%) followed by amytriptiline (17.9%) and beta-blockers (14.6%), whereas in episodic migraine the preferred options were beta-blockers (47.7%), topiramate (21.5%) and amytriptiline (13.4%).

Regarding efficacy perception, topiramate was considered the best option in chronic migraine (42.7%) followed by onabotulinumtoxinA (25.5%) and amitryptiline (22.4%). Considering episodic migraine, surveyed neurologists perceived topiramate (43.7%) and beta-blockers (30.3%) as the best options.

When evaluating the duration of the treatment in case of therapeutical response, in episodic migraine 43.5% of neurologists preferred 3 months and 39.5% 6 months, whereas in chronic migraine, 20.4% recommended 3 months, and 42.1%, 12.5% and 22.4% increased respectively to 6, 9 and 12 months.

When considering onabotulinumtoxinA therapy, the number of prior therapeutic failures was cero in 7.2% of neurologists, one in 5.9%, two in 44.1%, three in 30.9% and four or more in 11.9%. After a first ineffective procedure with Onabotulinumtoxin, 49% of responders affirmed not to increase the dose.

**Conclusions:**

Initial management of Migraine among Spanish Neurologists take into account the preventative drugs considered as first choices in most of the guidelines. Management of episodic migraine differed from chronic migraine, both in the preferences and the perception of efficacy.

**Introduction:**

Headache is the most frequent reason for referral to outpatient Neurology Offices in Spain^1^. Most neurologists have to deal with headache patients, being migraineurs the most frequent subgroup in our setting^2^. Spanish Society of Neurology periodically publishes guidelines on the main neurological conditions, in order to help clinicians in their daily practice.

Spanish Headache Study Group guidelines^3^ recommended Topiramate and Beta-blockers as the first choices of preventive therapy in Episodic Migraine, followed by Flunarizine, Lisinopril and Candesartan; only in a third level they recommended Valproic Acid, which use is currently contraindicated in women in fertile age^4^. On the other hand, the first oral preventive option in Chronic Migraine is Topiramate, followed by Amitriptyline and in a third level Flunarizine and Beta-blockers.

Despite the high number of therapies available, some of them have not been properly studied and the evidence is based on experts’ opinions^5^. The arrival of new drugs to our armamentarium should improve the care of migraine patients^6, 7^. Understanding which the needs of clinicians are might help in the elaboration of the new therapeutic strategies and guidelines.

We aimed to evaluate the preferences in the management of migraine with preventive therapies, and the perception about efficacy and safety of the current therapies among Spanish Neurologists.

**Material and methods:**

We conducted an observational, transversal study. The studied population was the community of Spanish Neurologists ascribed to the Spanish Society of Neurology.

The study included an anonymous survey mailed three times to all neurologist members of the Society. It included 8 questions about demography and 43 concerning preventative preferences and utilization.

In the analysis of the surveys we considered if the neurologist were a member of the Spanish Headache Study Group (SHSG) and those in which the headache disorders were the main area of interest. For analytic purposes, we categorized responders in two differentiated age groups: junior neurologists if they were younger than 40 years old (Juniors) and senior neurologists if they had 40 or more (Seniors in advance). In Spain the usual age when the Neurology specialization ends is 29, after 4 years of residency.

Demographic variables were gender, age group, region of origin, main area of interest, adscription to the SHSG, and time since they were focused on headache.

We considered as eligible drugs all the included in Official Guidelines of the Spanish Headache Study Group, including: beta-blockers, amitriptiline, topiramate, valproic acid, zonisamide, lisinopril, candesartan, fluoxetine, venlafaxine, desvenlafaxine, lamotrigine, magnesium, flunarizine, riboflavine, pregabalin, OnabotulinumtoxinA and anaesthetic blockade of occipital nerves^3^. We analyzed drugs individually, including two specific questions about the percentage of combined treatments in the first visit and in global.

We specifically evaluated which was the preferred beta-blocker and we included among the possible answers propranolol, nadolol, atenolol, bisoprolol, nebivolol and esmolol. We analyzed by separate OnabotulinumtoxinA (OnabotA) management in terms of number of prior preventive failures, employed units per procedure and long-term management.

Surveyed neurologists were asked about their usual first and second choices in preventive therapy in chronic migraine (CM), episodic migraine with aura (EMWA), episodic migraine without aura (EMOA). We also considered special conditions such as depression or pregnancy.

Participants were also questioned about their personal opinion considering the most effective drugs in both Chronic and Episodic Migraine and which they thought were the best-tolerated drugs.

The Scientific Committee of the Spanish Society of Neurology approved the study and all the participants agreed to participate voluntarily.

We present data as number and percentage. Missing data was managed by complete case analysis. We employed SPSS v16.0 for the Statistical Analysis. For the comparison of qualitative variables, we used Chi^2^ test. In the comparison of continuous variables with qualitative variables, the employed test was Student t test and Median test in case of non-normal distribution or <30 variables per group. Correlation between quantitative variables was analysed with Pearson test. We considered an alpha error value of 0.05.

**Results:**

*Demographic parameters:*

We received answers from 153 neurologists, among which 53 (34.9%) were ascribed to the Spanish Headache Study Group. The percentage of female participants was 43.4%. Age of participants was < 30 years in 11.8%, 30-39 in 41.4%, 40-49 in 16.4% and >50 in 30.3%.

The most frequent regions were Madrid, with 32 participants (21.1%) and Catalonia with 24 (15.8%). The time since they were focused to headache disorders was <6 years in 35.4%, 6-10 years in 14.6%, 11-15 years in 14.6%, 16-20 in 10.4% and >20 years in 25%.

***First choice drugs:***

*First choice in chronic migraine:*

Topiramate was the first choice drug in 57% of responders, followed by Amitriptyline (17.8%), Beta-blockers (14.6%) and Flunarizine (6%). The most frequent second choice drug were Beta-blockers (25.7%) followed by Topiramate (23.2%), Amitriptyline (20.5%), OnabotulinumtoxinA (10.6%) and Flunarizine (9.3%). Full data about all the possible drugs is shown in Supplementary material.

Management of CM seemed to be different among general neurologists when compared with those focused on headache disorders, specially concerning the election as first choice drug of topiramate (49.5% vs. 71.2%), amitriptyline (23.2 vs 7.7%) and flunarizine (9.1% vs 0%). (p:0,017).

When compared between juniors and seniors we did not found significant differences.

*First choice in episodic migraine without aura:*

The preferred drugs were Beta-blockers (47.7%), followed by Topiramate (21.5%), Amitriptyline (13.4%) and Flunarizine (11.4%). The most frequent second choice was Topiramate (44.6%), followed by Beta-blockers (23.6%), Amitriptyline (15.5%), Flunarizine (9.5%) and Zonisamide (4.1%). Further data is available in Supplementary materials.

*First choice in episodic migraine with aura:*

Topiramate was the first choice for 50.3% of participants, followed by Beta-blockers (23.2%) Amitriptyline (9.3%), Flunarizine (6.6%) and Lamotrigine (2.6%). The most frequent second choices were Topiramate (31.8%), Beta-blockers (25.7%), Lamotrigine (10.1%), Amitriptyline (9.5%), Zonisamide (8.8%), Flunarizine (6.8%) and Valproic Acid (6.1%). Full list of answers is available in Supplementary materials.

We did not found significant differences between Headache Specialists and general neurologists concerning the preferred drug in episodic migraine treatment, neither with nor without aura.

Between junior and seniors, there was a trend to use less Topiramate among Juniors (17.7% vs. 25.7%) and Flunarizine (8.9% vs. 14.3%) and use more Amitriptyline (16.5% vs. 10.0%) (p:0,1).

Figure 1 represents the pooled first and second choices in Chronic Migraine (CM), Episodic Migraine with aura (EMWA) and Episodic Migraine without aura (EMOA).

Management was described to be different when treating female patients in 76.3% of neurologists. The preferred option in case of pregnancy was Beta-blockers in 57.7%, Magnesium (17.3%), Greater Occipital Nerve Blockades (16.3%), Onabotulinumtoxin (3.8%), Lamotrigine (2.9%) and others (2%). Valproic acid was avoided in female migraineurs by 90.1% of responders.

In patients with depression, the preferred drugs were Amitriptyline in 67.1%, Venlafaxin in 15.3%, and Topiramate in 11.3%.

**Responses about perception of efficacy:**

*Chronic migraine:*

Topiramate was considered the most effective drug in the treatment of CM by 42.7% of responders, followed by OnabotulinumtoxinA in 25.2%, Amiptriptyline (22.4%), Beta-blockers (3.5%) and Flunarizine (2.1%).

The second most frequently considered drug was also topiramate in 34.3%, followed by Amitriptyline (19.7%), Beta-blockers (15.3%), OnabotulinumtoxinA (8%), Venlafaxin (5.8%), Valproic Acid and Zonisamide (5.1% each). Full list of responses can be consulted in Supplementary materials.

Responders with Special Interest in Headache disorders considered OnabotulinumtoxinA as the most effective drug in (34%) of the cases compared with general Neurologists (18.2%) (p:0,029), and General Neurologists selected Amitriptyline as the most effective option in 27.3% compared with 9.4% among Headache Specialists (p:0.10). We did not found statistically significant differences between junior and senior neurologists.

*Episodic migraine:*

The drug perceived as most effective in episodic migraine was Topiramate (43.7%), followed by Beta-blockers (30.3%), Amitriptyline (14.8%) and Flunarizine (8.5%). Beta-blockers were considered the second most effective option in 32.9% of the answers, followed by Topiramate (28.6%), Amitriptyline (17.1%) and Flunarizine (11.4%). Full data about efficacy can be consulted in Supplementary materials.

Preferences of Neurologists with special interest of headache did not differed from General Neurologists, only Amitriptyline was described as less effective (17.6% vs. 25.0%, but differences were not statistically significant p:0,56).

We found differences in the perception of efficacy of Topiramate and Amitryptiline comparing Senior and junior neurologists. Seniors considered Topiramate as the most effective more often (54.5%vs34.2%, p:0,022),whereas juniors considered Amitriptyline as the most effective in more cases (21.1%vs7.6%, p:0,033).

The preferred beta-blocker among surveyed neurologists was propranolol (90, 62.1%), followed by nadolol and nebivolol (23, 15.9% each), atenolol (6, 4.1%) and bisoprolol (3, 2.1%).

**Perceptions about tolerability:**

The drugs considered as the best tolerated in a young patient without any comorbidity were betablockers (42.4%), followed by flunarizine (14.6%), OnabotulinumtoxinA (11.3%), topiramate and amitriptyline (9.3% each), and magnesium (4%). Full data about tolerability preferences can be seen in Supplementary Materials.

Figure 2 shows survey responses considering tolerability, showing the percentage of neurologists that considered each drug as the best tolerated and the second best tolerated respectively.

Opinion about the best-tolerated drug tended difference in Headache Specialists when compared with general neurologists but we did not reach statistical signification. Figure 3.

In the correlation analysis we found that the correlation between considering Topiramate good tolerated and selecting it as first choice in CM was r=0,95, (p=0,24, Pearson test), in EMOA was r=0,23 (p=0,005) and in EMWA r=0,23 (p=0,005); whereas the correlation between considering it as the most effective and selecting it as first choice was higher, r=0,46 in EMOA (r<0,0001) and r=0,43 in EMWA (p<0,0001) and for CM was r=0,31 (p<0,0001).

**Management of patients:**

Participants described patients comorbidities as the most important factor when choosing a preventative (70.2%) followed by guidelines recommendations (13.9%), personal experience (11.3%) and patients preferences (4.6%).

Regarding the management in primary care, 80.9% of the surveyed neurologists considered that the general practitioner should prescribe preventatives before referral to neurology office. The number of preventatives considered to fail before referring the patient was none in 4.6%, one in 13.8%, two in 56.6% and three or more in 25% for episodic migraine whereas in chronic migraine was none in 21.1%, one in 22.4%, two in 40.8% and three or more in 15.8%.

Regarding the duration of preventive therapy when a response was achieved, in EM 51% of responders affirmed to maintain the treatment 3 months or less whereas in CM 42,1% treated during 6 months and 34,9% during at least 9 months. Figure 4 shows the duration of the treatment in EM or CM.

Politherapy was only considered in selected cases in the first visit by 22.3% of the participants. When asked about management in their patients, the percentage of them treated with polytheraphy was estimated to be 0-10% by 14.5% of neurologists, 11-25% in 44.1%; 26-50% in 27.6%; 51-75% in 9.2% and >76% in 4.6%.

**OnabotulinumtoxinA management:**

Regarding specific OnabotA questions, we asked about the number of failure (efficacy or tolerability) in oral preventatives before starting OnabotA. Response was none in 7.2% of neurologists, one in 5.9%, two in 44.1%, three in 30.9%, four in 9.9% and five or more in 2%. When asked whether they considered specifically the failure to any preventative, 51.4% mentioned topiramate, 4.9% amitryptiline, 4.2% beta-blockers and 36,6% affirmed they did not consider any specific failure.

When comparing between headache specialists and general neurologists, the first group started OnabotA earlier than general neurologists (p:0.014). Figure 5.

We did not found differences when comparing Juniors and Seniors and 54.3% affirmed to start OnabotA after two preventive failures and 83,9% after the failure of three preventive drugs (p:0.49).

Concerning OnabotA dose, in the first procedure, 50.9% of responders affirmed to inject 155 Units and 41.7% 150U. *Figure 6* shows the units used per procedure by the surveyed neurologists. After a first ineffective procedure, 51% of responders increased the dose in the second procedure and in case of inefficacy, 83% of responders did increased the dose from 155 units in the third procedure.

The number of OnabotA procedures before considering it as ineffective was two in 18.9% of neurologists, three in 70.8% and four in 10.4%.

Only 66 participants (38,2%) described to be assisted by a nurse in the OnabotA preparation. The percentage of clinicians that self-charged the medication was higher among Headache Specialists compared with general neurologists (50.9 vs. 39.4%, p:0.008).

Figure 7 shows the percentage of patients in which responders affirmed to perform a reduction of OnabotulinumtoxinA in case of efficacy, trying to stop the therapy and figure 8 shows the percentage in which they were able to finally stop it.

**Discussion:**

*First choice:*

The main findings of our study were that Topiramate was selected as the first choice drug in CM and EMWA and the second choice in EMOA, whilst Beta-blockers were the drugs of choice in EMOA. These findings follow the Spanish Headache Study Group recommendations^3^, but differences were found in the second and third choice, as few responders considered Valproic Acid as a potential option and Amitriptyline was selected before Flunarizine in all three groups.

According to European Medicines Agency^4^ recommendations, subscribed by the European Headache Federation, Valproic Acid should be avoided in women in fertile age. In our sample, 90.1% of responders followed that recommendation.

The percentage of responders that mentioned other drugs such as Candesartan^8^, Lisinopril^9^, Zonisamide^10^, Lamotrigine^11^ was around 10%, despite our guidelines place some of them in the same level as Amitriptyline.

*Efficacy:*

Only Topiramate and OnabotA have proved their efficacy in randomized controlled trials in Chronic Migraine and only recently its efficacy has been directly compared^12^. Despite the different methodology of the studies, number of headache days reduction in the pivotal trials ranged from -3,5 to -6,4 for Topiramate^13, 14^and -7,8 to -9 for OnabotulinumtoxinA^15,16^. Our findings suggest that neurologists perceived Topiramate as more effective than OnabotulinumtoxinA, both among Headache Specialists and General Neurologists. In the last group, perception about OnabotulinumtoxinA efficacy was even lower and Amitryptiline was chosen as more efficacious.

We also consider of interest the perception about Beta-blockers, as they were considered as the second most effective option in EM, slightly after Topiramate. However, in Chronic Migraine, the percentage of neurologists that considered them as the most effective option was significantly lower, only 3.5% them in comparison with 30.3% in EM. Only one study compared face to face them and could not found statistically significant differences in Episodic Migraine patients^17^.

Another topic that can be matter of discussion is the duration of the preventive treatment. Spanish guidelines recommend at least 6 months of treatment^3^, but we found a significant heterogeneity. We should harmonize the minimal duration, how much should we increase the dose in case of lack of tolerability and how long should we keep the treatment before considering it a failure.

*Tolerability:*

Concerning the tolerability profile, most of the responders preferred Beta-blockers but the preferred drugs were Flunarizine, OnabotulinumtoxinA, Amitriptyline and Topiramate. Some of these drugs have been associated with adverse events in up to 75 to 82,5% of the patients in the pivotal trials^13, 14^.

Despite of a different way of administration, OnabotulinumtoxinA was perceived as well tolerated by a high percentage of neurologists, in line with long-term studies show^18^, and it was selected before GON blockades.

*OnabotulinumtoxinA:*

Spanish experts recommendations for the use of OnabotulinumtoxinA stated that the first procedure should be performed according to the PREEMPT paradigm, administering 155 Units and in case of lack of response, dose could be increased up to 195 Units in the first three procedures^19^. We found that in certain cases, neurologists affirmed to use lesser or higher doses.

To date, two factors that have been associated with an increased efficacy to OnabotA in CM are the shorter evolution of Chronic Migraine and a lesser number of oral preventatives prior to OnabotA using^20^. Despite that Spanish Guidelines recommend to consider OnabotA after two therapeutic failures and Spanish Health Care System covers OnabotulinumtoxinA costs all across the country, only 49.5% of general neurologists surveyed affirmed to start it after the failure of two preventatives, similar data as in an Italian survey, showing that only 39,7% of responders affirmed to start before the failure of 3 preventatives^21^. Publication of European guidelines^22, 23^ should encourage clinicians to consider it when indicated as it has proven to be an effective therapy also in real world studies^20^.

*Future perspectives:*

The fact that all currently available preventive drugs have been developed for other indications might be related with the presence of adverse events and premature interruption of the treatment. The arrival of novel specific drugs such as anti-CGRP antibodies and Gepants could change dramatically the clinical picture^24, 25^. Nevertheless, despite most of the participants affirmed that the presence of comorbidities was the main factor in the selection of the therapy; we found that the decision was more correlated with the perception of efficacy rather than the tolerability profile.

The panorama will change in the following years and some factors will be of striking importance, such as the availability of the therapies, its efficacy in real world setting and the diffusion and perception for the majority of clinicians. We support the creation of new European Guidelines and the harmonization of the National Guidelines according to the current literature.

Our study has some limitations. As an online survey, not all the members of the Headache Study Group responded to it and the answers might not represent the opinions of all the Headache Specialists. Among the 51 questions, some of them were subjective and we did not allowed more than two possible answers.

Also, our results reflect only opinion among Spanish neurologists, with a Public Healthcare System different of those from many other European Countries.

The conclusions of our study state that the main criteria in the selection of treatments was the subjective perception of efficacy, being Topiramate the drug considered as the most effective and therefore the first choice drug in CM and EMWA. Despite the availability of many novel therapies, most of the clinicians employed the classical drugs.

**BIBLIOGRAPHY:**

1. Matías-Guiu JA, García-Azorín D, García-Ramos R et al. Study of outpatient neurological care in the Region of Madrid: The impact of implementing free choice of hospital. Neurología 2015;30: 479-487.
2. Pedraza MI, Mulero P, Ruíz M, et al. Characteristics of the first 2000 patients registered in a specialist headache clinic. Neurología. 2015;30:208–213.
3. Ezpeleta D, Pozo-Rosich P. Guías diagnósticas y terapéuticas de la Sociedad Española de Neurología. Guía oficial de práctica clínica en cefaleas. Madrid. Editorial Luzan 5. 2015, vol. 53.
4. Vatzaki E, Strauss S, Dogne JM et al. Latest clinical recommendations on valproate use for migraine prophylaxis in women of childbearing age: overview from European Medicines Agency and European Headache Federation. J Headache Pain. 2018;19(1)68.
5. Linde M, Mulleners WM, Chronicle EP, McCrory DC. Topiramate for the prophylaxis of episodio migraine in adults. Cochrane Database of Systematic Reviews2013, Issue 6.
6. Martelletti P. The application of CGRP® Monoclonal antibodies in migraine spectrum: Needs and priorities. BioDrugs 2017(6)483-485.
7. Negro A, Curto M, Lionetto L, et al. Chronic migraine treatment: from OnabotulinumtoxinA onwards, Exp Rev Neurotherap 2016(10)1217-1227.
8. Trovnik E, Stovner LJ, Helde G et al. Prophylactic treatment of migraine with an angiotensin II receptor blocker: a randomized, controlled trial. JAMA 2003;289(1):65-69.
9. Schrader H, Stovner LJ, Helde G et al. Prophylactic treatment of migraine with angiotensin converting enzyme inhibitor (Lisinopril): randomised, placebo controlled, crossover study. BMJ 2001;322:19-22.
10. Pascual-Gómez J, García-Naya M, Leira R. et al. Zonisamide in the preventive treatment of refractory migraine. Rev Neurol 2010;50:129-132.
11. Steiner TJ, Findley LJ, Yuen AW. Lamotrigine versus placebo in theprophylaxis of migraine. Cephalalgia 1997;17:109-112.
12. Rothrock JF, Adams AM, Jo E, et al. A Multicenter, Prospective, Randomized, Open-Label Study to Compare the Efficacy, Safety, and Tolerability of OnabotulinumtoxinA and Topiramate for Headache Prophylaxis in Adults with Chronic Migraine: The FORWARD Study. Neurology 2018;90, supplement P4.134.
13. Silberstein SD, Lipton RB, Dodick DW et al. Efficacy and safety of topiramate for the treatment of chronic migraine: a randomized, double-blind, placebo-controlled trial. Headache 2007;47:170-180
14. Diener H-C, Bussone G, Van Oene JC et al. Topiramate reduces headache days in chronic migraine: a randomized, double-blind, placebo-controlled study. Cephalalgia 2007;27:814–23. doi:10.1111/j.1468-2982.2007.01326.x.
15. Aurora SK, Dodick DW, Turkel CC et al. OnabotulinumtoxinA for treatment of chronic migraine: Results from the double-blind, randomized, placebo-controlled phase of the PREEMPT 1 trial. Cephalalgia 2010;30:793–803.
16. Diener HC, Dodick DW, Aurora SK et al. OnabotulinumtoxinA for treatment of chronic migraine: Results from the double-blind, randomized, placebo-controlled phase of the PREEMPT 2 trial. Cephalalgia 2010;30:804–14.
17. Ashtari F, Shaygannejad V, Akbari M. A double-blind, randomized trial of low-dose topiramate vs propranolol in migraine prophylaxis. Acta Neurol Scand 2008;118:301-305.
18. Blumenfeld AW, Stark RJ, Freeman MC et al. Long-term efficacy and safety of OnabotulinumtoxinA for the prevention of chronic migraine: COMPEL study. J Headache Pain. 2018;19(1):13.
19. Sarchielli P, Romoli M, Corbelli I et al. Stopping onabotulinum treatment after the first two cycles might not be justified: Results of a real-life monocentric prospective study in chronic migraine. Front Neurol 2017;8,655.
20. Dominguez C, Pozo-Rosich P, Torres-Ferrús M et al. OnabotulinumtoxinA in chronic migraine: predictors of response. A prospective multicentre descriptive study. Eur J Neurol 2018;25:411-416.
21. Tasorelli C, Aguggia M, De Tomasso M et al. Onabotulinumtoxin A for the management of chronic migraine in current clinical practice: results of a survey of sixty-three Italian Headache Centers. J Headache Pain. 2017;18(1): 66.
22. Bendtsen L, Sacco S, Ashina M. Guideline on the use of OnabotulinumtoxinA in chronic migraine: a consensus statement from the European Headache Federation. J Headache Pain 2018;19-91.
23. Gago-Veiga AB, Santos-Lasaosa S, Cuadrado ML et al. Evidence and experience with onabotulinumtoxinA in chronic migraine: Recommendations for daily clinical practice. Neurología. 2017; S0213-4853.
24. Reuter U. A review of monoclonal antibody therapies and other preventative treatments in migraine. Headache 2018;58 suppl 1:48-59.
25. Bigal ME, Rappaport AM, Silberstein SD. From LBR-101 to Fremanezumab for Migraine. CNS Drugs 2018;32:1025-1037.

**Figures legends:**

Figure 1:

First choice drugs in Chronic Migraine (CM) (blue), Episodic Migraine With Aura (EMWA) (red) and Episodic Migraine Without Aura (EMOA) (green). Percentage shows the pooled percentage of people who responded each drug as first or second choice in each indication.

Figure 2:

Best tolerated drugs according to responders’ opinions. In light blue, percentage of responders that selected each drug as the best tolerated. In light orange, percentage of responders that selected each drug as the second best tolerated. Number represents percentage.

Figure 3:

Opinions among surveyed neurologists about each drug tolerability. In orange: answers from Neurologists with special interest in headache. In green: answers from general neurologists.

Figure 4:

Optimal duration of preventative treatment according to surveyed neurologists. Numbers represent the percentage of responders that answered each duration. Orange bars represent episodic migraine; red bars represent chronic migraine.

Figure 5:

Number of oral preventative failures prior to the OnabotulinumtoxinA therapy.Numbersrepresent the percentage of responders that affirmed to start OnabotulinumtoxinA after the failure of each number of failures. In orange, answers from Headache Specialists; Green, answers from general neurologists.

Figure 6:

Percentage of responders that affirmed to administer each number of OnabotulinumtoxinA units in the first (light blue), second (dark blue) or third (purple) procedure.

Figure 7:

Percentage of responders that try to stop OnabotulinumtoxinA therapy when it is effective.

Figure 8:

Percentage of responders that affirm to be able to stop OnabotulinumtoxonA therapy when it is effective.

SUPPLEMENTARY MATERIALS:

Supplementary material 1:

First and second choices in Chronic Migraine.Number represent percentage or the total responses.

| Chronicmigraine | Firstchoice | Secondchoice |
| --- | --- | --- |
| Betablockers | 14,6 | 25,7 |
| Amitryptiline | 17,8 | 20,5 |
| Topiramate | 57 | 23,2 |
| Valproicacid | 0 | 2,6 |
| Zonisamide | 1,3 | 3,9 |
| Lisinopril | 0 | 0 |
| Candesartan | 0 | 0,7 |
| Fluoxetin/venlafaxin | 0 | 1,3 |
| Lamotrigin | 0 | 0 |
| Magnesium | 0,7 | 0 |
| Flunarizine | 6,0 | 9,3 |
| Riboflavine | 0,7 | 0,7 |
| Pregabaline | 0 | 0,7 |
| OnabotulinumtoxinA | 2 | 10,6 |
| GON blockade | 0 | 0 |

Supplementary materials 2: First and second choices in Episodic Migraine Without Aura. Numberrepresentspercentage of total responses.

| EM OA | Firstchoice | Secondchoice |
| --- | --- | --- |
| Betablockers | 47,7 | 23,6 |
| Amitryptiline | 13,4 | 15,5 |
| Topiramate | 21,5 | 44,6 |
| Valproicacid | 0,7 | 1,4 |
| Zonisamide | 0 | 4,1 |
| Lisinopril | 0 | 0 |
| Candesartan | 0,7 | 0 |
| Fluoxetin/venlafaxin | 0 | 0 |
| Lamotrigin | 0 | 0 |
| Magnesium | 3,4 | 0 |
| Flunarizine | 11,4 | 9,5 |
| Riboflavine | 0,7 | 0 |
| Pregabaline | 0 | 0 |
| OnabotulinumtoxinA | 0 | 0,7 |
| GON blockade | 0,7 | 0,7 |

Supplementary materials 3: First and second choices in Episodic Migraine With Aura. Numbers represents percentage of total responses.

| EMWA | Firstchoice | Secondchoice |
| --- | --- | --- |
| Betablockers | 23,2 | 25,7 |
| Amitryptiline | 9,3 | 9,5 |
| Topiramate | 50,3 | 31,8 |
| Valproicacid | 2,6 | 6,1 |
| Zonisamide | 2,0 | 8,8 |
| Lisinopril | 0,7 | 0 |
| Candesartan | 0 | 1,4 |
| Fluoxetin/venlafaxin | 0 | 0 |
| Lamotrigin | 2,6 | 10,1 |
| Magnesium | 1,3 | 0 |
| Flunarizine | 6,6 | 6,8 |
| Riboflavine | 0,7 | 0 |
| Pregabaline | 0 | 0 |
| OnabotulinumtoxinA | 0 | 0 |
| GON blockade | 0,7 | 0 |

Supplementary material 4: Perception about efficacy of the different drugs in the treatment of Chronic Migraine. Numbers represent percentage of the answers.

| Effective CM | Mosteffective | Secondmosteffective |
| --- | --- | --- |
| Betablockers | 3,5 | 15,3 |
| Amitryptiline | 22,4 | 19,7 |
| Topiramate | 42,7 | 34,3 |
| Valproicacid | 2,1 | 5,1 |
| Zonisamide | 1,4 | 5,1 |
| Lisinopril | 0 | 0,7 |
| Candesartan | 0 | 0,7 |
| Fluoxetin/venlafaxin | 0,7 | 5,8 |
| Lamotrigin | 0 | 0 |
| Magnesium | 0 | 0,7 |
| Flunarizine | 2,1 | 4,4 |
| Riboflavine | 0 | 0 |
| Pregabaline | 0 | 0,7 |
| OnabotulinumtoxinA | 25,2 | 8 |
| GON blockade | 0 | 0 |

Supplementary material 5: Perception about efficacy of the different drugs in the treatment of Episodic Migraine. Numbers represent percentage of the answers.

| Effective EM | Mosteffective | Secondmosteffective |
| --- | --- | --- |
| Betablockers | 30,3 | 32,9 |
| Amitryptiline | 14,8 | 17,1 |
| Topiramate | 43,7 | 28,6 |
| Valproicacid | 0,7 | 3,6 |
| Zonisamide | 1,4 | 2,1 |
| Lisinopril | 0 | 0 |
| Candesartan | 0 | 0 |
| Fluoxetin/venlafaxin | 0 | 0,7 |
| Lamotrigin | 0 | 0 |
| Magnesium | 0 | 0,7 |
| Flunarizine | 8,5 | 11,4 |
| Riboflavine | 0 | 0 |
| Pregabaline | 0 | 0 |
| OnabotulinumtoxinA | 0 | 1,4 |
| GON blockade | 0,7 | 1,4 |

Supplementary material 6: Answers about the drugs considered as the best tolerated and second best tolerated.

| Tolerated | Besttolerateddrug | Secondbesttolerated |
| --- | --- | --- |
| Betablockers | 42,4 | 23,8 |
| Amitryptiline | 9,3 | 28,7 |
| Topiramate | 9,3 | 9,1 |
| Valproicacid | 2 | 4,9 |
| Zonisamide | 2 | 6,3 |
| Lisinopril | 0 | 0,7 |
| Candesartan | 1,3 | 4,2 |
| Fluoxetin/venlafaxin | 0 | 2,8 |
| Lamotrigin | 0,7 | 1,4 |
| Magnesium | 4 | 2,1 |
| Flunarizine | 14,6 | 10,5 |
| Riboflavine | 2,6 | 0 |
| Pregabaline | 0 | 0 |
| OnabotulinumtoxinA | 11,3 | 2,8 |
| GON blockade | 0,7 | 2,8 |
